# Creep Deformation and Its Effect on Mechanical Properties and Microstructure of Magnesium Phosphate Cement Concrete

**DOI:** 10.3390/ma16051760

**Published:** 2023-02-21

**Authors:** Yuxin Gao, Jihui Qin, Zhen Li, Xingwen Jia, Jueshi Qian

**Affiliations:** College of Materials Science and Engineering, Chongqing University, Chongqing 400044, China

**Keywords:** magnesium phosphate cement concrete, shrinkage, creep, mechanical properties, microstructure

## Abstract

Creep deformation is an important aspect of magnesium phosphate cement (MPC) used as a structural material. In this study, the shrinkage and creep deformation behaviors of three different MPC concretes were observed for 550 days. The mechanical properties, phase composition, pore structure, and microstructure of MPC concretes after shrinkage and creep tests were investigated. The results showed that the shrinkage and creep strains of MPC concretes stabilized in the ranges of −140 to −170 με and −200 to −240 με, respectively. The low water-to-binder ratio and the formation of crystalline struvite were responsible for such low deformation. The creep strain had almost no effect on the phase composition; however, it increased the crystal size of struvite and reduced the porosity, especially the volume of pores with diameters <20 nm and >200 nm. The modification of struvite and densification of microstructure led to an improvement in both compressive strength and splitting tensile strength.

## 1. Introduction

Magnesium phosphate cement (MPC) is a kind of clinker-free cement, also known as chemically bonded magnesium phosphate ceramic, which is prepared mainly by blending dead-burned magnesia (MgO) with acid phosphate [1]. Ammonium dihydrogen phosphate (NH_4_H_2_PO_4_) [2,3] and potassium dihydrogen phosphate (KH_2_PO_4_) [4,5,6,7,8,9] are the most commonly used acid phosphates for fabricating MPC. The acid-base neutralization reaction between MgO and phosphate to form struvite or k-struvite is the principal driving force of MPC’s microstructure and performance development [5,10,11,12]. Generally, the principal hydration and hardening reaction of MPC can be described using Equations (1) or (2):

(1) $${\mathrm{MgO}+\mathrm{NH}}_{4}H_{2}{\mathrm{PO}}_{4}{+5H}_{2}O\;\to {\;\mathrm{NH}}_{4}{\mathrm{MgPO}}_{4}{\cdot 6H}_{2}O\;(\mathrm{struvite})$$

(2) $${\mathrm{MgO}+\mathrm{KH}}_{2}{\mathrm{PO}}_{4}{+5H}_{2}O\;\to {\;\mathrm{KMgPO}}_{4}{\cdot 6H}_{2}O\;(k\text{-}\mathrm{struvite})\;$$

Struvite or k-struvite provides cementitious properties for the MPC system. When compared with ordinary Portland cement (OPC) and other inorganic binders, MPC shows an array of merits because of its unique reaction mechanisms and microstructural characteristics. These include rapid setting [13,14], low alkalinity [15], high early and long-term strength [3,13], low drying shrinkage [3,16], excellent bonding properties [17], good salt corrosion resistance [18], and fire resistance [19]. Due to these properties, MPC becomes very attractive in a wide range of applications, including (but not limited to) rapid repairing and reinforcement of existing concrete structures (e.g., bridges, buildings) [14,20,21,22], coating materials for steel and concrete [19,20,21], and potential binders for conditioning and encapsulating nuclear wastes [20,21,23]. Recent research also involved its use in biomaterials due to its good biocompatibility and antibacterial properties [24].

Despite the recognition of MPC, it still has several important properties that have not been studied adequately, for instance, shrinkage and creep behaviors. Some experimental studies have been conducted on the free shrinkage of MPC materials. MPC develops very low shrinkage at an early age when compared to OPC, as shown by several studies [15,16]. Li [25] reported that the 28-day drying shrinkage of MPC mortar only corresponded to 10–25% of that of OPC concrete. These investigations mainly focused on the short-term shrinkage of MPC pastes or mortars (within 1 year). However, for massive structures applications, the study of long-term shrinkage of MPC concrete is equally essential.

In addition to shrinkage, creep is also a particularly important aspect of MPC used as a structural material. Creep is the deformation of concrete under sustained loading, which may lead to an excessive deflection of the concrete structure and even cause severe serviceability problems [26,27]. MPC concrete generally exhibits much higher strength than OPC concrete at an early age [14]. This allows MPC concrete to have an earlier loading time in structural applications, such as prefabricated buildings and prestressed engineering. In the case of prestressed concrete structures, creep must be known in advance since creep is used to calculate the prestressing losses, which is important for the safety of concrete structures [28]. In addition, creep loading can significantly affect the long-term characteristics of concrete [29]. However, scarce information is available on the creep performance of MPC concrete and its influence on long-term microstructure and properties.

In this context, this paper focuses on studying the long-term deformation of MPC concretes, where three MPC concretes with different contents and gradations of coarse aggregates were designed, and their drying shrinkage and creep were monitored for 550 days under natural (indoor) curing conditions. The mechanical strengths, phase composition, pore structure and microstructure of MPC concretes after shrinkage and creep test were investigated to evaluate the influence of creep strain on the mechanical and microstructural properties. This work may be the first experimental study on the creep of MPC concrete, which will provide preliminary knowledge of the long-term deformation of MPC concrete under sustained stress.

## 2. Materials and Methods

### 2.1. Raw Materials

**Figure 1:**
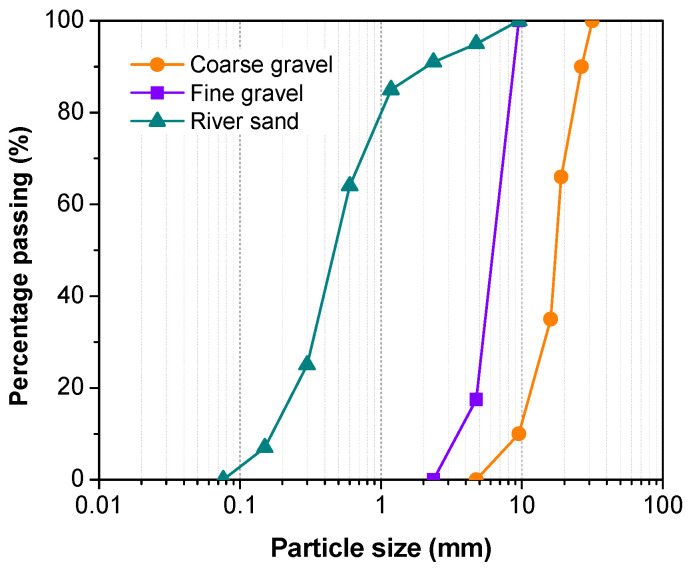
Particle size distributions of river sand and gravel.

Commercial MPC was supplied by Guizhou Linmei Materials Co., Ltd., Guiyang, China. The main components of this cement were dead-burned MgO, ammonium dihydrogen phosphate (NH_4_H_2_PO_4_), and borax decahydrate (Na_2_B_4_O_7_·10H_2_O). F-class fly ash (FA) obtained from a local coal-fired thermal power station, with a surface area of 3850 cm^2^/g and a density of 2.80 g/cm^3^, was incorporated into the binder. The chemical compositions of MPC and FA are shown in Table 1. The aggregates used in this study were natural sand with a specific gravity of 2.58 and fine/coarse gravel with a specific gravity of 2.61. The maximum particle size of natural sand, fine gravel, and coarse gravel were 4.75, 9.5, and 26.5 mm, respectively. The cumulative sieve residue curves of natural sand and gravel are shown in Figure 1.

### 2.2. Concrete Mixture and Sample Preparation

The mix proportions of MPC concrete are shown in Table 2. Three MPC concrete mixes designated as M1, M2, and M3 were fabricated. In all the mixes, the FA replacement level in MPC was 10 wt.%, the water-to-binder (w/b) ratio of all formulations was fixed at 0.17, and the ratio of sand to gravel was kept at 0.5. M2 had higher binder content and lower aggregate content in comparison to M1. In the production of M3, the amount of coarse gravel was reduced, and the content of fine gravel was increased in comparison to M2.

The ingredients were weighed in designated proportions and then thoroughly mixed for 3 min in a single horizontal-axis-forced concrete mixer after adding water. The fresh mix was poured into specific molds and then vibrated for 30 s on the vibration table. After 3 h of casting, the specimens were demolded and further cured in a laboratory (indoor environment) until the testing age.

### 2.3. Test Methods

**Figure 2:**
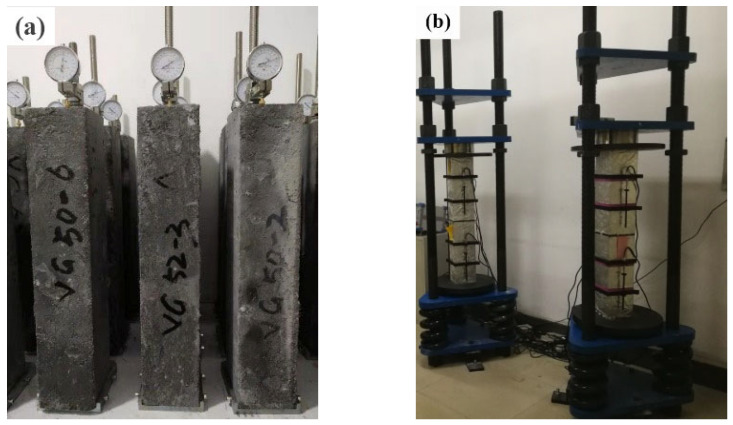
Test for (**a**) shrinkage and (**b**) creep of MPC concrete.

Three identical prismatic specimens with dimensions of 100 mm × 100 mm × 515 mm were prepared for each mix and used to measure unrestrained shrinkage following GB/T 50082-2009 [30]. These shrinkage specimens are also referred to as “unloaded” specimens or as MX-S series (where MX represents M1, M2, or M3). Prepared shrinkage specimens were vertically placed in the shrinkage test frame and a dial gauge was mounted on the top of each prism, as shown in Figure 2a. The first dial gauge readings were recorded 3 days after molding. Thereafter, specimen lengths were periodically measured for 550 days. The shrinkage test was performed in a laboratory room. The shrinkage strains over time were calculated by using Equation (3). The final shrinkage strain value of each mix reported was averaged from three specimens.

(3) $$\varepsilon_{\mathrm{st}}{=(L}_{0}-L_{t})/L_{b}$$

where *ɛ*_st_ represents shrinkage strain at the age of t (day), *L*_b_ = 500 mm represents the effective length of the specimen that eliminates the embedded length of the copper head in the top end, *L*_0_ represents the initial length of the specimen (mm) and *L*_t_ represents the length measured at the age of t (day) (mm).

The prism strength of concrete is essential for investigating compressive creep under a certain stress–strength ratio. The compressive strength was measured from 100 mm × 100 mm × 300 mm prisms, as per GB/T 50081-2019 [31]. The creep was determined on 100 mm × 100 mm × 400 mm prisms 3 days after molding, according to GB/T 50082-2009 [30]. The applied compressive stress corresponded to 40% of the average 3-day prismatic compressive strength. These creep specimens are also referred to as “loaded” specimens or as MX-C series (where MX represents M1, M2, or M3). For each creep test, two specimens were stacked in the creep test frame for simultaneous and sustained loading, as shown in Figure 2b. Longitudinal strains were simultaneously recorded on two sides of each prism using deformation gauges with a gauge length of 100 mm. The creep measurement continued for a period of 550 days under natural conditions. Equation (4) was used to determine the creep strain at a certain time.

(4) $$\varepsilon_{\mathrm{ct}}=(\Delta L_{t}-\Delta L_{0})/L_{\mathrm{cb}}-\varepsilon_{\mathrm{st}}$$

where *ɛ*_ct_ represents the creep strain of concrete at the age of t (day), Δ*L*_t_ represents total deformation value at the age of t (day) (mm), Δ*L*_0_ represents initial deformation (mm), *L*_cb_ represents the gauge length (mm) and *ɛ*_st_ is unrestrained shrinkage strain measured from unloaded companion specimens at the age of *t* (day).

After the shrinkage and creep tests, the prismatic specimens were cut into 10 cm cubes for mechanical tests according to GB/T 50081-2019 [31]. The loading rate was 1 MPa/s for the compressive strength test, and 0.25 MPa/s for splitting tensile strength measurement.

Fractured samples were collected from the core of the specimens after the mechanical tests. They were soaked in absolute ethanol for 24 h to stop hydration, and then vacuum dried at 40 °C for another 24 h. Subsequently, the samples were ground and sieved to less than 75 μm before X-ray diffraction (XRD) and thermogravimetric (TG) analysis. Block samples with a size of 3–5 mm were selected for scanning electron microscopy (SEM) and mercury intrusion porosimetry (MIP) tests.

The XRD test was performed with a Panalytical X-Pert Pro X-ray diffractometer (Netherlands). The continuous scanning mode of 3°/min was used in the 2θ range of 10–80°. The TG test was carried out in a nitrogen atmosphere using a Netzsch TG 209 F3 analyzer (Germany) at a heating rate of 5 °C/min up to 600 °C. The pore structure was characterized by using a Micromeritics’ Autopore IV 9500 instrument (United States). The test pressure was in the range of 0.518 to 32,923.57 psi, corresponding to the pore size range of 348.63 μm to 5.5 nm. The morphology of the fracture surface of selected samples was observed by using ZEISS Gemini 300 scanning electron microscope (Germany). Before the test, the sample was sprayed with gold by Quorum SC7620 sputtering coating instrument (United Kingdom) under vacuum.

## 3. Results and Discussion

### 3.1. Shrinkage

**Figure 3:**
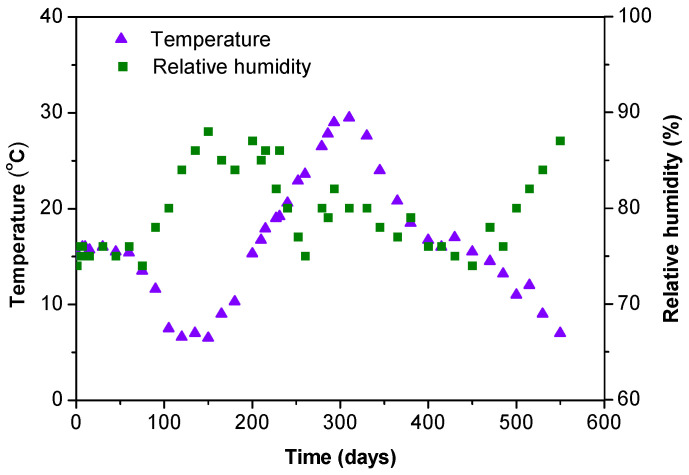
Changes in ambient temperature and relative humidity during the test period.

MPC concrete specimens were placed in a laboratory without constant temperature and relative humidity. The changes in temperature and relative humidity during the test period are shown in Figure 3. At the beginning of the test (before 75 days), the temperature was maintained at 15 ± 2 °C and the relative humidity of interior air was stabilized between 74–76%. Afterward, the temperature and relative humidity changed with the seasons. The maximum temperature was nearly 30 °C in summer and the minimum was approximately 6 °C in winter. The relative humidity of indoor air increased as the temperature dropped, and exceeded 80% when the temperature was lower than 10 °C. The deformation behaviors of MPC concrete could be affected by ambient temperature and relative humidity.

**Figure 4:**
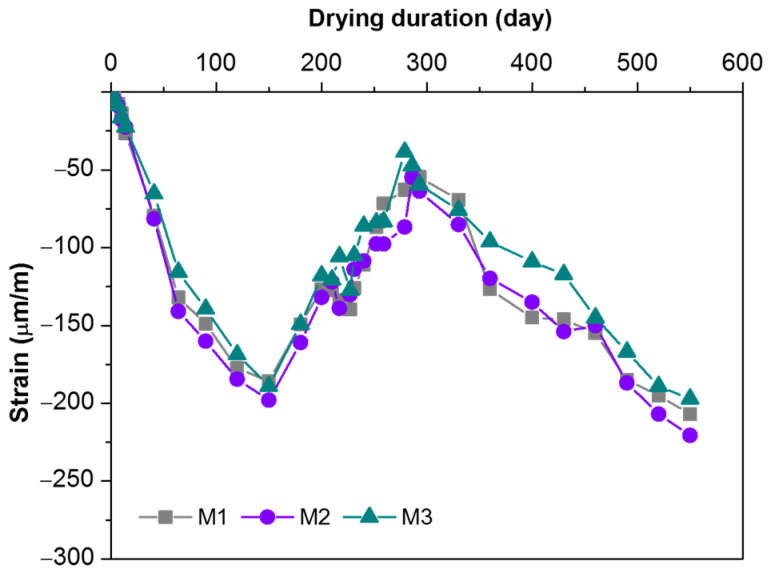
The measured total strain of unloaded MPC concrete specimens.

Measured strains from unloaded specimens with different aggregate contents and gradation are shown in Figure 4. In general, all samples revealed a similar trend of strain development, regardless of the mix proportions. The measured strains from unloaded specimens are shrinkage strains. The shrinkage strain increased firstly and reached −185 to −200 με after around 150 days, and then decreased to −40 to −55 με after around 280 days. Afterward, it continued to increase from −200 to −220 με at the end of the test. The significant fluctuation of measured strains was consistent with the variation of temperature with the seasons. This suggested that the measured strains from unloaded MPC concrete specimens contained unignorable thermal strains.

The strains measured from unloaded specimens included thermal strain, which is attributable to the significant change in temperature. The thermal strain can be calculated using Equation (5).

(5) $$\varepsilon_{\mathrm{tt}}{=\alpha }_{c}{(T}_{t}-T_{0})$$

where $\varepsilon_{\mathrm{tt}}$ is the thermal strain at the age of *t*(day), $\alpha_{c}$ = 8.4 × 10^–6^/°C = 8.4 με/°C is the coefficient of thermal expansion of MPC concrete according to the previous literature [16], *T*_t_ is the daytime temperature at the age of *t*(day) (°C), and *T*_0_ = 15 °C is the initial temperature value. After removing the thermal strain, the shrinkage strain corresponds to the one measured at *T*_0_.

**Figure 5:**
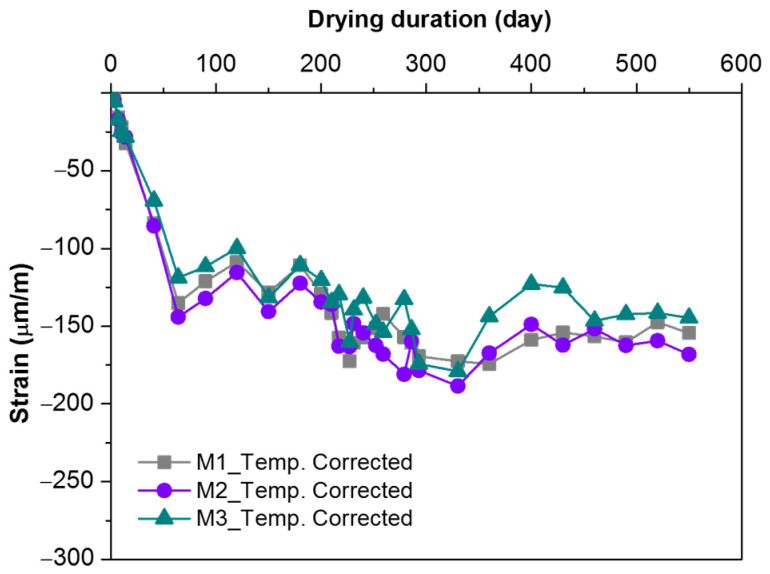
The measured temperature-corrected strain of unloaded MPC concrete specimens.

Figure 5 shows the temperature-corrected shrinkage strains of unloaded specimens. The shrinkage strains showed a continuous increase at the beginning of the test and reached −120 to −135 με after around 65 days. It was estimated that the shrinkage strain was mainly due to the decreased content of free water as a result of its participation in reaction in the early stage and migration or evaporation in the late stage [6]. After 65 days, the shrinkage strain continuously fluctuated (probably due to changes in relative humidity) and tended to be stable until the end of the test. The final temperature-corrected shrinkage of MPC concrete reached −140 to −170 με, which corresponded to one-third of that of Portland cement [32]. M2 had a slightly higher shrinkage strain than M1 or M2 due to its higher cement content. The w/b ratio of MPC concrete in this study was only 0.17, which was much lower than that of Portland cement concrete with the same strength grade [33,34]. Excess water in concrete exists in the form of free water. When free water is lost, capillary pressure increases and shrinkage occurs; therefore, the more free water there is, the greater the drying shrinkage is.

### 3.2. Creep

**Figure 6:**
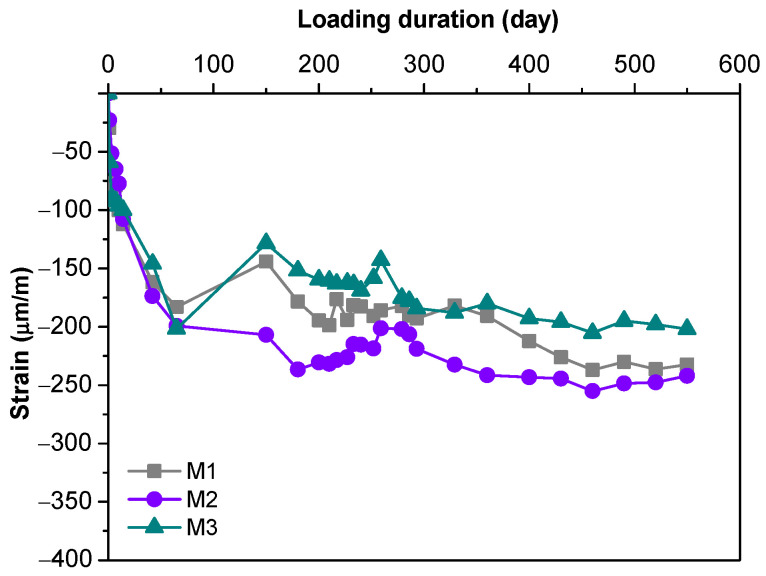
Measured total strain of loaded MPC concrete specimens.

Creep strains of MPC concretes with different mix proportions under sustained compression are presented in Figure 6. They were obtained by subtracting elastic and shrinkage deformation values from the measured total strains. In general, similar trends were observed for all samples. The creep strains increased rapidly during the early test period and reached −180 to −200 με after 65 days. Within this test period, ambient temperature and relative humidity remained relatively stable (see Figure 3). This implies that the measured strains from loaded specimens did not contain thermal strain. Creep strains began to gently fluctuate after 65 days and the fluctuation tended to be stable at the end of the test, indicating that creep strains did not change much when temperature and relative humidity varied significantly between day 65 and 550.

The final creep strains of MPC concretes stabilized between −200 and −240 με after 550 days. However, the creep strain of OPC concrete with the same strength level generally reaches around −500 με [35], which is more than twice the value of MPC concrete. This may be due to the fact that the major binding phase of Portland cement is calcium silicate hydrate (C-S-H) gel [36], while the principal hydration products of MPC are crystalline struvite. The water-facilitated and time-dependent natures of the texture development of crosslinked C-A-S-H provide new insights into the mechanism of creep in cement-based materials. The gradual preferential re-orientation of C-A-S-H nano-crystallites induced by sustained stress could account for the creep development, while portlandite and ettringite with a limited degree of texture formation would contribute less to the creep of cement-based materials [37]. Struvite in MPC concrete generally showed a smooth texture [38] and high crystallization grade, which can be considered positive for an enhancement of creep deformation.

Creep is the result of plastic deformation between hardened cement paste and aggregates, which is introduced by the viscoelasticity of cement paste in concrete and the interlayer slip of hydrated phases [39]. The role of aggregates is to restrain the deformation of hydrated cement paste; therefore, the more hydration products, the greater the creep [40]. The cement content of M2 was higher than in M1, leading to the higher creep value of M2. M3 exhibited a lower creep than M2, suggesting that the creep can be reduced when the aggregate gradation is optimized.

### 3.3. Mechanical Properties

**Figure 7:**
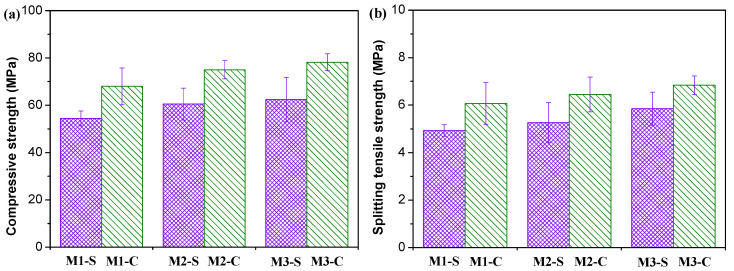
Mechanical properties of MPC concrete samples after 550 days of shrinkage and creep testing. (**a**) compressive strength; (**b**) splitting tensile strength.

The mechanical properties of MPC concrete specimens, which had been loaded for 550 days, and of unloaded specimens are shown in Figure 7. The loaded specimens revealed higher mechanical properties than the unloaded specimens. The compressive strength and splitting tensile strength of the creep specimens were increased by 24.0–25.3% and 16.9–23.1%, respectively. The increase in compressive strength was also reported for loaded conventional concrete at a loading level of 40% [41], and alkali-activated slag concrete at loading levels of 35% and 50% [42]. However, the results published by Liniers [43] showed that tensile strength drop occurs after a compressive load above 40% of the compressive strength since micro-cracking takes place under such load. It is believed that the effect of creep on the mechanical properties of concrete strongly depends on several conditions, including loading levels, loading age, and loading direction [29]. In this work, the load was applied after 3 days with a loading level of 40%. The positive effect of creep may be related to the modification of the hydration product and/or the densification of microstructures [43] under moderate compressive loads. This will be further discussed in the following sections. In addition, the creep can change the internal stress distribution [44], thus making the stress of concrete more uniform and reasonable and reducing the risks of stress concentration when encountering failure load.

It could be observed that the content and graduation of coarse aggregate have a slight influence on the strength. The order of the compressive strength values of shrinkage and creep specimens with different contents and graduation of coarse aggregate was M1 < M2 < M3 (Figure 7a). The splitting tensile strength shown in Figure 7b had a similar trend to the compressive strength of MPC concretes during the sustained loading or unconstrained shrinking. M1 had a relatively lower strength than M2 due to its lower cement content. At the same cement content, the aggregate gradation of M3 was better than in M2, which can explain the higher strength of the former. Increasing the amount of cement can improve strength but increase shrinkage and creep. The optimization of aggregate gradation enhanced both mechanical properties and shrinkage and creep deformation.

### 3.4. Microstructure Analysis

#### 3.4.1. XRD Analysis

**Figure 8:**
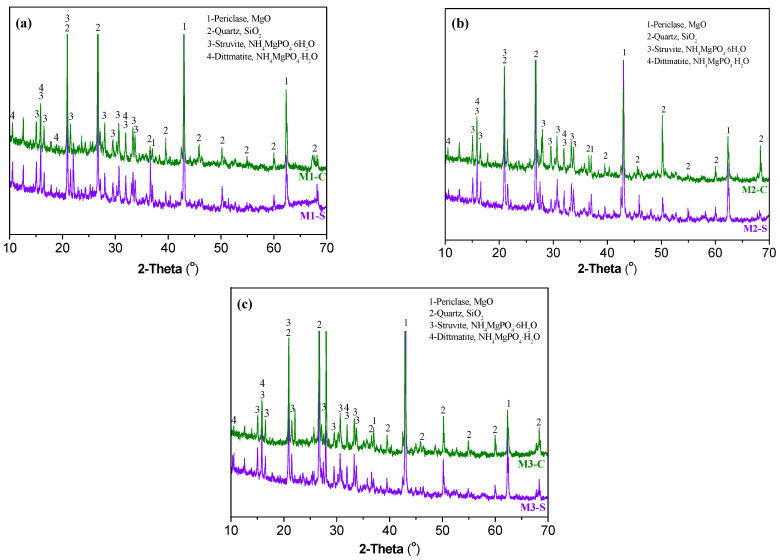
XRD patterns of MPC concrete samples after 550 days of shrinkage and creep tests. (**a**) M1, (**b**) M2, and (**c**) M3.

**Figure 9:**
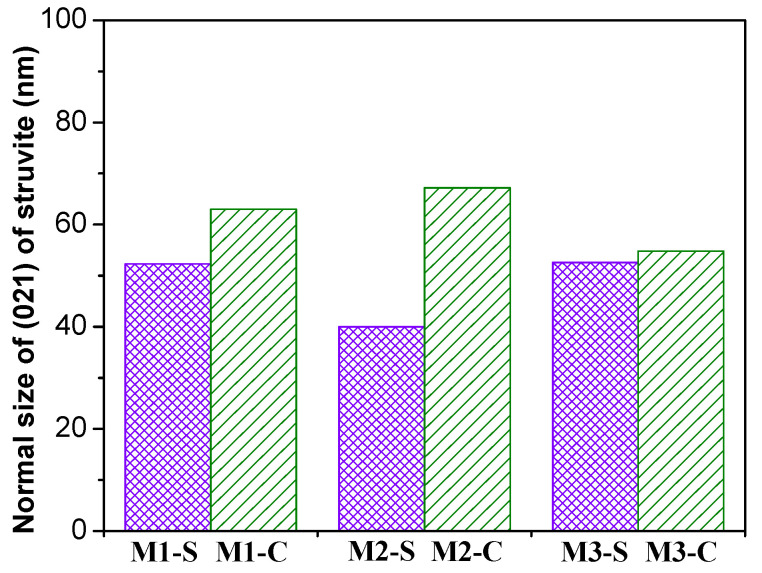
The crystal size of struvite [021] of MPC concrete specimens.

The XRD patterns of MPC samples after shrinkage and creep tests are shown in Figure 8. Comparison of these patterns shows that the crystalline phases of the samples were consistent after the creep and shrinkage tests, mainly including unreacted periclase (MgO), quartz (SiO_2_), struvite (NH_4_MgPO_4_·6H_2_O), and dittmarite (NH_4_MgPO_4_·H_2_O). Unreacted periclase, identified as MgO, was generally added in excess to ensure the complete reaction of all phosphates; it could act as micro-aggregates and thus restrain the deformation of hydration products. Struvite was founded to be the main crystalline product of MPC, which has cementitious properties and provides excellent mechanical properties for MPC concrete. The minor presence of dittmarite could be from the reaction between MgO and NH_4_H_2_PO_4_ or the dehydration of struvite at a peak hydration temperature higher than 70 °C [13]. These results indicate that the sustained loading did not change the type of hydration products. However, the sustained compressive stress could change the grain size of struvite. The crystal size of struvite [021] is calculated using Equation (6) as recommended by [45], and the results are presented in Figure 9. The calculations are based on the assumption that the broadened width of the diffraction peak is only caused by the size of the crystal structure, which is uniform. It is shown that the crystal size of struvite was increased after 550 days of sustained loading. The increase in crystal size of struvite may change the internal stress distribution and improve the mechanical properties of MPC concrete.

(6) D =Kλ/βcosθ

where *D* is the crystal size (nm), K = 0.89 is Scherrer constant; *λ* represents the wavelength of X-ray (nm); *β* is the half-width of the diffraction peak (rad); *θ* is the diffraction angle (rad).

#### 3.4.2. Thermal Analysis

**Figure 10:**
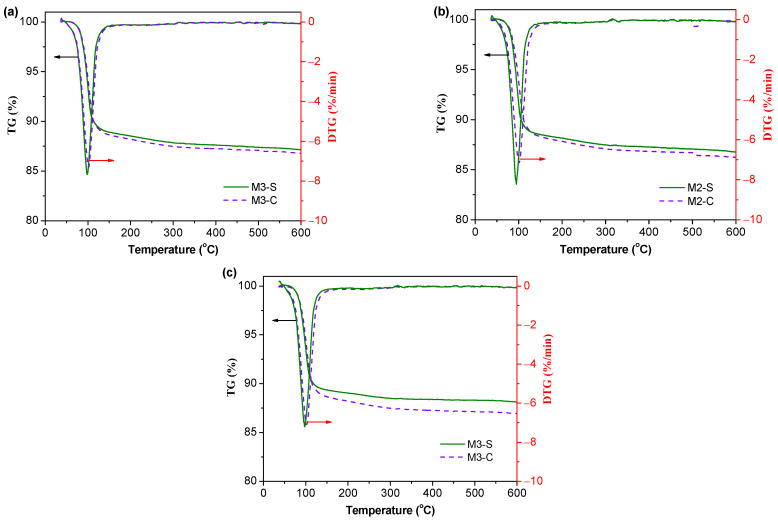
TD-DTG curves of MPC concrete samples after 550 days of shrinkage and creep tests. (**a**) M1, (**b**) M2, and (**c**) M3.

The TG curves and differential (DTG) curves of MPC samples after shrinkage and creep tests are shown in Figure 10. Only one weight loss event between 50 and 200 °C was observed in the curves, which was associated with the decomposition of struvite [46]. This result further confirms that creep strains do not affect the type of hydration products. When compared with shrinkage samples, creep samples revealed higher weight losses observed up to 200 °C. For example, the weight losses below 200 °C were 10.9% and 11.8% for M3-S and M3-C, respectively. This strongly suggests that sustained stress can promote the formation of struvite, which has a positive effect on mechanical strengths. A similar result was confirmed by Liu [47], who highlighted that compression load would help cement-based material, especially at an early age, to promote hydration.

#### 3.4.3. Analysis of Pore Structure

**Figure 11:**
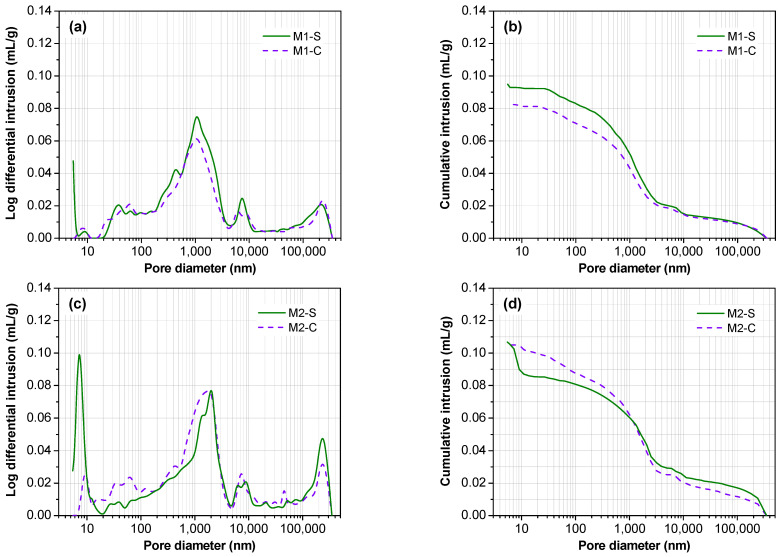

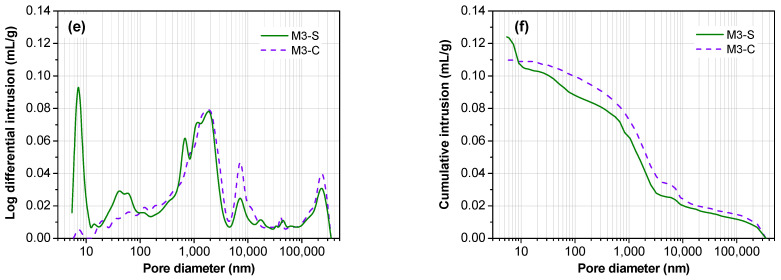
Pore size distribution of different MPC concrete samples. (**a**) log differential intrusions of M1, (**b**) cumulative pore volume of M1, (**c**) log differential intrusions of M2, (**d**) cumulative pore volume of M2, (**e**) log differential intrusions of M3, (**f**) cumulative pore volume of M3.

Figure 11 and Table 3 show the pore size distribution and the total porosity of different MPC concrete samples after 550 days of shrinkage and creep tests. As shown in Figure 11, the cumulative mercury intake of M2 and M3 was slightly higher than in M1. This is because M2 and M3 had higher cement content and thus higher reaction degrees between cement components and water, which generates more pores. The sustained stress can reduce the cumulative amount of mercury, implying that the reduction of porosity is a key factor in the generation of creep strain under long-term load pressure. In general, the porosity of cement-based materials is inversely proportional to their strength. Compared with the shrinkage specimens, the creep specimens exhibited a slightly denser microstructure with a smaller porosity and thus higher compressive strength and splitting tensile strength.

To further clarify the differences in pore structure between shrinkage and creep specimens, the pore size was divided into four categories according to previous studies [48], i.e., harmless pores (<20 nm), less harmful pores (20–100 nm), harmful pores (100–200 nm) and more harmful pores (>200 nm). The creep strain will reduce the volume of harmful and more harmful pores, to improve the strength. On the other hand, the amount of harmless and less harmful pores was significantly reduced as well after creep testing. This may be related to the increase in the crystal size of struvite, as described in XRD results. Although creep reduced the total porosity, it improved the median pore and average pore diameter. The increase in median pore and average pore size was closely related to the decreased volume of harmless pores. In addition, creep samples exhibited higher tortuous complexity of pore structure in comparison to shrinkage samples. Higher complexity of concrete pore structure generally leads to a higher strength, which partially explains the improvement of compressive strength and splitting tensile strength in creep specimens.

#### 3.4.4. SEM Microstructure Analysis

**Figure 12:**
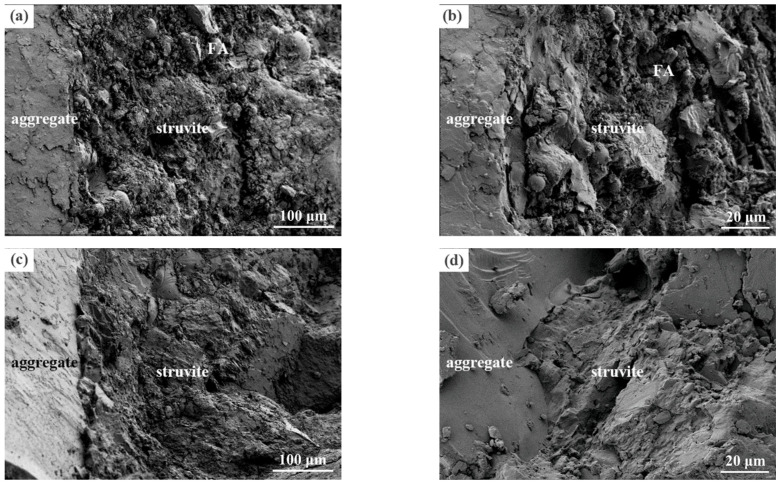
Typical microstructures of fracture surfaces of selected samples after creep and shrinkage tests. (**a**) shrinkage sample (×500), (**b**) shrinkage sample (×2000), (**c**) creep sample (×500), and (**d**) creep sample (×2000).

Typical microstructures of fracture surfaces of selected samples after creep and shrinkage tests are shown in Figure 12. Some particles with round shapes were observed in the samples due to the incorporation of FA. Abundant struvite crystals with less regular morphology were formed, constructing a dense microstructure. It is reported that a loose interface transition zone between aggregate and hardened MPC paste was observed at an early age due to wall effect [49]. However, it can be seen from Figure 12 that the interface between aggregate and hydrated cement paste was closely combined after shrinkage and creep tests. By comparing these figures, no obvious differences can be found in the microstructures of the fracture surface of shrinkage and creep samples.

## 4. Conclusions

This study investigated the shrinkage and creep deformation behaviors of MPC concretes under natural curing conditions, as well as their mechanical properties and microstructural changes after shrinkage and creep tests. The following conclusions can be drawn:

(1) Under natural conditions, temperature changes affected significantly the free shrinkage strain but had less effect on the compressive creep strain. The shrinkage and creep strains of MPC concretes stabilized in the ranges of −140 to −170 με and −200 to −240 με after 550 days, respectively, highlighting the good volume stability of MPC concrete. This can be attributed to the low water-to-cement ratio and the formation of crystalline struvite;
(2) The creep strain had almost no effect on the type of hydration products but increased the crystal size and the quantity of hydration product struvite. The microstructure of MPC concrete can be densified under sustained loading, characterized by reduced porosity, particularly in the volume of pores smaller than 20 nm and larger than 200 nm;
(3) Compared to shrinkage specimens, the compressive strength and splitting tensile strength of creep specimens were increased by 24.0–25.3% and 16.9–23.1%, respectively. The positive effect of creep on the mechanical strengths of MPC concrete could be due to the modification of the hydration products and densification of the microstructure.

## Figures and Tables

**Table 1:** Chemical compositions of MPC and FA (wt.%).

Compound	MgO	P_2_O_5_	SiO_2_	Al_2_O_3_	Fe_2_O_3_	CaO	TiO_2_	Na_2_O	SO_3_	K_2_O	MnO
MPC	57.85	17.01	11.80	6.36	2.20	1.26	1.04	0.78	0.45	0.26	0.18
FA	1.32	-	50.30	27.78	6.08	5.01	1.99	-	1.05	-	-

**Table 2:** The mix of MPC concrete per unit volume (kg/m^3^).

Sample	MPC	FA	Sand	Coarse Gravel	Fine Gravel	Water
M1	585	65	600	1200	0	110
M2	630	70	580	1160	0	119
M3	630	70	580	780	380	119

**Table 3:** Total porosity and pore size distribution of different MPC concrete samples.

Sample	Total Porosity (%)	Pore Size Distribution (%)				Average Pore Diameter (nm)	Median Pore Diameter (nm)	Tortuosity
		Harmless Pores (<20 nm)	Less Harmful Pores (20–100 nm)	Harmful Pores (100–200 nm)	More Harmful Pores (>200 nm)			
M1-S	18.77	0.28	2.68	1.03	14.79	133.9	8.9	67.78
M1-C	17.06	0.91	1.85	0.77	13.53	179.4	35.7	88.10
M2-S	21.23	4.80	1.06	0.74	14.63	38.1	8.3	9.70
M2-C	20.35	1.30	2.45	0.86	15.73	128.4	16.5	14.81
M3-S	24.53	0.69	3.69	0.95	19.20	41.5	8.1	15.95
M3-C	20.94	0.13	1.84	1.05	17.92	251.3	34.5	26.88

## Data Availability

Not applicable.
